# Nutritional Properties and Oxidative Indices of Broiler Breast Meat Affected by Wooden Breast Abnormality

**DOI:** 10.3390/ani10122272

**Published:** 2020-12-02

**Authors:** Krittaporn V. Thanatsang, Yuwares Malila, Sopacha Arayamethakorn, Yanee Srimarut, Nantawat Tatiyaborworntham, Tanaporn Uengwetwanit, Atikorn Panya, Wanilada Rungrassamee, Wonnop Visessanguan

**Affiliations:** National Center for Genetic Engineering and Biotechnology (BIOTEC), Pathum Thani 12120, Thailand; krittaporn.tha@ncr.nstda.or.th (K.V.T.); sopacha.ara@biotec.or.th (S.A.); yanee.sri@biotec.or.th (Y.S.); nantawat.tat@biotec.or.th (N.T.); tanaporn.uen@biotec.or.th (T.U.); atikorn.pan@biotec.or.th (A.P.); wanilada.run@biotec.or.th (W.R.); wonnop@biotec.or.th (W.V.)

**Keywords:** commercial broiler, wooden breast, breast meat, meat quality, protein oxidation, absolute gene expression, droplet digital polymerase chain reaction

## Abstract

**Simple Summary:**

Chicken breast is considered as a good source of high-quality protein and essential trace minerals. However, the nutritive quality of the meat can be adversely affected by wooden breast (WB) myopathy. WB manifests as decreased contents of total protein (per gram of meat) and essential amino acids isoleucine, leucine and valine. In addition, the mineral profile of WB meat is abnormal. The cause of WB remains unclear, but it has been linked with oxidative stress within the breast muscle in the living birds. In this study, protein oxidation in the meat and changes in absolute expression of oxidative stress response genes were identified, strengthening the link between oxidative stress and the incidence of WB.

**Abstract:**

Wooden breast (WB) abnormality adversely impacts the quality of chicken meat and has been linked with oxidative stress. In this study, breast samples were taken from carcasses of 7-week-old Ross 308 broilers 20-min and 24-h postmortem. Five WB and seven non-WB control samples were assigned based on palpatory hardness (non-WB = no unusual characteristics and WB = focal or diffused hardness). WB exhibited lower contents of protein and the amino acids, i.e., isoleucine, leucine and valine, lighter surface color, lower shear force, greater drip loss and altered mineral profiles (*p* ≤ 0.05). Despite no difference in lipid oxidation, a greater degree of protein oxidation was found in the WB meat (*p* ≤ 0.05). Absolute transcript abundances of superoxide dismutase, hypoxia inducible factor 1 alpha and pyruvate dehydrogenase kinase 1 were greater in WB (*p* ≤ 0.05), whereas lactate dehydrogenase A expression was lower in WB (*p* ≤ 0.05). The findings support an association between oxidative stress and the altered nutritional and technological properties of chicken meat in WB.

## 1. Introduction

Chicken meat, particularly the breast portion, is widely recognized as an inexpensive source of high-quality protein providing adequate amounts of all of the essential amino acids with a protein digestibility corrected amino acid score value ranging between 0.91 and 0.95 [1]. Among different land animal meats, chicken breast has low lipid and collagen contents, the latter of which is more favorable for digestibility [2,3]. Poultry meat is also an important source of minerals, in particular iron, zinc, copper and selenium [3].

In response to consumer demand, modern broilers (meat-type chickens), have been intensively selected through a breeding program for an accelerated growth rate and larger breast mass. The success in breeding selection, however, has coincided with an increased occurrence of chicken myopathies, including wooden breast (WB) syndrome [4,5]. WB is characterized by hardened ridges extending from the cranial to the caudal regions, pale color and surface hemorrhagic lesions present on the pectoralis major muscle [6,7,8], WB abnormality is a global concern in the poultry industry as development of this myopathy alters the appearance of the meat and its physicochemical and technological properties [9,10,11,12]. The average prevalence of the myopathy has been reported at 10% per broiler flock [11,13]. The likelihood of WB incidence positively correlates with the proportion of breast mass relative to total broiler carcass weight, supporting a potential association between WB development and artificial breeding selection for fast-growing broilers [11]. Enlarged breast muscle fibers may exhibit inadequate vascularization [14,15], which limits oxygen availability and metabolic waste removal leading to an accumulation of reactive oxygen species (ROS) within the breast muscle. Accumulation of ROS can trigger oxidative stress within the muscle fibers [16]. Oxidative stress within WB muscle has been reported from studies of differential gene expression [17,18,19,20] and metabolomic profiling [21,22], suggesting a possible etiology for WB.

It is widely known that ROS as primary initiators of oxidative processes can readily react with other biomolecules, particularly lipids, proteins and DNA [23]. Disruption of muscle cells can promote lipid oxidation, causing unpleasant flavor and meat discoloration due to oxidation of heme pigment [24]. Lipids consisting of highly unsaturated fatty acids, including omega-3 and omega-6, are particularly susceptible to oxidation. The oxidative degradation of polyunsaturated essential fatty acids, therefore, reduces both shelf-life and the nutritive value of meat [23,25]. Moreover, a variety of lipid oxidation products are considered to be risk factors for human health [26]. ROS can also induce protein carbonylation either directly via homolytic cleavage of C-H bonds or indirectly through reactions with the carbonyl group-containing lipid oxidation products. Upon oxidation, polypeptide backbone and amino acid side chains, in particular aromatic and sulfhydryl groups, are modified. Protein oxidation can induce fragmentation, polymerization and aggregation of proteins, which consequently reduce the functional properties and nutritional quality of the proteins [27].

Previous studies of WB focused on reduced technological properties of the affected breasts [8,11,28] as this issue directly exerts a considerable economic impact on the poultry and food processing industries. However, the etiology of WB is still under an extensive investigation. In this study, the amino acid profiles and lipid and protein oxidation between WB-affected and unaffected breast samples were compared. The quality indices and absolute expression of four key genes in the oxidative stress response were also elucidated.

## 2. Materials and Methods 

### 2.1. Sample Collection and Characterization of Muscle Abnormalities

The breast samples used in this study were immediately collected from the carcasses of male 7-week-old Ross 308 broilers within 20 min postmortem at a local slaughterhouse (Saraburi, Thailand). The breasts were classified as “WB” or “non-WB” based on the consistency and palpation scoring system of Sihvo et al. [29]. The WB samples (*n* = 5) used in this study exhibited a markedly hardened consistency, which was consistent with the breasts graded as “WB1hard” in the study of Sihvo et al. [29]. The non-WB controls (*n* = 7) did not exhibit any defective characteristics. 

Within 20 min of the sample collection, one of the breasts (pectoralis major) from each carcass was cut into small cubes and snap frozen in liquid nitrogen. The snap-frozen samples, labeled as “skeletal muscle”, were stored at −80 °C until analysis of physicochemical properties and gene expression. The other side of the breast samples, labeled as “meat sample”, was kept in a plastic bag at 2 °C until 24 h postmortem. The meat samples were then used for examining the effects of WB condition on chemical composition, oxidation of lipid and protein and technological properties of the broiler breast meat.

All samples were purchased in the form of whole carcasses from the commercial processing plant. No experimental treatments or scientific procedures were conducted on living animals. Therefore, ethical approval for animal experimentation is not required.

### 2.2. Properties of 20-min Postmortem Skeletal Muscle

The initial pH value (pH_20_), glycogen and lactate contents of the muscle samples were analyzed as described previously [20]. For the pH_20_ measurement, 1 g of pulverized muscle sample was homogenized with 10 mL iodoacetate buffer (pH 7.0) containing 5 mM sodium iodoacetate and 150 mM potassium iodoacetate and the pH of the homogenate was then measured using a pH meter (Mettler-Toledo Seven Easy, Mettler-Toledo, Inc., Greifensee, Switzerland). Lactate content was determined using an L-lactic acid assay kit (Megazyme Ltd., Wicklow, Ireland). The lactate was extracted from the muscle (500 mg) according to the manufacturer’s recommendation for the muscle tissue sample. The measurement was done in duplicate. Lactate concentration was determined from the supernatant and expressed in milligrams per gram of muscle sample. A glycogen assay was conducted, in duplicate, on 20-min postmortem muscle samples as described previously [20]. Crude glycogen was first extracted from the muscle using the method previously described [21]. The extracted glycogen was then measured using a glycogen assay kit (Sigma-Aldrich, St. Louis, MO, USA) and expressed in milligrams per gram of muscle sample. 

### 2.3. Chemical Composition, Oxidation of Lipid and Protein and Technological Properties of 24-h Postmortem Breast Meat

Properties of the 24-h postmortem meat samples were analyzed as follows. First, surface color of each breast meat sample in the CIE L*, a* and b* system was determined using a Minolta color meter (model CR300, Minolta Co. Ltd., Osaka, Japan), at three designated areas, i.e., the cranial, middle and caudal regions after the sample was allowed to bloom for 30 min at 4 °C. The color difference (∆E) between non-WB and WB samples was calculated from the following formula: ∆E = [(∆L*)^2^ + (∆a*)^2^ +(∆b*)^2^]^1/2^. The ultimate pH (pHu) was then determined by directly inserting a spear-shaped pH probe into the same regions assigned for color determination. The cranial end was then cut into 3-inch-wide samples, which were used for determination of the water holding capacity and texture of cooked meat. The remaining meat was then homogeneously ground and used for determining chemical composition, lactate content and lipid and protein oxidation.

The proximate composition, including moisture, crude protein, crude fat and ash, of the meat samples was determined according to the Association of Official Analytical Chemists (AOAC) standard methods [30]. Following the ash determination, elements and trace elements in the ash were profiled using an Orbis PC Micro-x-ray fluorescence (XRF) analyzer (EDAX, Inc., Mahwah, NJ, USA). Ash samples (100 mg) were pressed between two layers of 4-µm thick polycarbonate film and placed in a micro-XRF sample holder. Elemental profiling was accomplished under vacuum conditions using an x-ray beamline at 30 KV and 1000 µA. The acquisition time was 60 s for single point analysis on the prepared pellets with the x-ray tube radius of 1 mm and 1 mm penetration depth. Data from each sample were acquired from five different areas and matched against the library provided with the Orbis Vision software (EDAX). The signal intensity of each element was reported using the Orbis Vision software (EDAX). The concentration of each element was calculated from linear calibration plots obtained by measurement of the absorbance of standard solutions. The content of each mineral was converted to milligram per kilogram of meat. 

Amino acid profiles of the breast samples were determined using an Agilent 7890B GC/7000D gas chromatography–mass spectrometer (GC–MS) triple quadrupole system (Agilent Technologies, Santa Clara, CA, USA) following the method describes by Jimenez-Martín et al. [31] with a slight modification. In brief, 40 mg of the ground samples were hydrolyzed in 5 mL of 6 N HCl at 110 °C overnight. The neutralized hydrolysates were subsequently evaporated completely and derivatized with N-tert-butyldimethylsilyl-N-methyltrifluoroacetamide with 1% tert-butyldimethylchlorosilane (Sigma-Aldrich, St. Louis, MO, USA) and acetonitrile at 100 °C for 4 h. The derivatized samples (2 μL) were subsequently separated through an HP5 capillary column (30 m × 0.25 mm initial diameter × 0.25 µm thickness, Agilent Technologies) using GC–MS as described previously [31]. The MS spectra were recorded in a selected ion monitoring (SIM) mode. A mixture of amino acids with known concentrations was prepared and used as a standard to generate calibration curves. The amino acid content was calculated and expressed as a percentage of total amino acids. 

Lactate content in meat samples was analyzed in the similar manner conducted with the muscle samples. Lipid oxidation was determined as thiobarbituric acid reactive substances (TBARS) according to Buege and Aust [32] with modifications. Ground sample (2 g) was homogenized with 10 mL of TBARS reagent containing 26 mM thiobarbituric acid, 0.92 M trichloroacetic acid (TCA) and 0.25 M HCl using a homogenizer (model ULTRA-TURRAX^®^ T 25, IKA Werke, Staufen, Germany) at 11,000 rpm for 30 s. The colorimetric reaction was carried out by incubating the mixture in a boiling bath for 10 min. After cooling the sample container with running tap water, the homogenate was centrifuged at 3600× *g* for 25 min at 25 °C. The supernatant was measured spectrophotometrically at 532 nm against a reagent blank. TBARS values were calculated using a standard curve of 1,1,3,3, tetraethoxypropane, which is converted to malondialdehyde upon heating, and expressed as μmol malondialdehyde per kilogram of sample. 

Protein oxidation was determined, in duplicate, as protein carbonyls using a protein carbonyl assay kit (Sigma-Aldrich, St. Louis, MO, USA). Protein from the breast samples was extracted as described by Soglia et al. [33] with modifications. One gram of ground breast tissue was homogenized with 10 mL of 0.15 M KCl for 30 s. Protein in the homogenate (0.1 mL) was precipitated with 1 mL of ice-cold acetone and centrifuged at 3500× *g* for 2 min. After discarding the supernatant, the pellet was resolubilized in 0.4 mL of 5% (w/v) sodium dodecyl sulfate (SDS) by incubating on a ThermoMixer C (Eppendorf, Hamburg, Germany) at 100 °C with a constant shaking at 1500 rpm for 10 min. The SDS-solubilized protein (0.2 mL) was incubated with 0.1 mL of the 2,4-dinitrophenylhydrazine reagent for 10 min at room temperature according to the kit manufacturer’s procedure. The protein concentration was determined using a Pierce™ bicinchoninic acid protein assay kit (Thermo Fisher Scientific, Inc., Rockford, IL, USA). Bovine serum albumin was used as a protein standard. The protein carbonyl content (nmol/mg protein) was calculated using a millimolar extinction coefficient at 375 nm of 22 mM^−1^ cm^−1^.

Drip loss and cook loss were used as indicators of water holding capacity (WHC). The raw breast meat was individually packed in a plastic bag and hung at 4 °C for 24 h. Drip loss was expressed as the difference, in percentage, between the initial and final weights. The meat was vacuum-packed in a plastic bag and subsequently cooked by water immersion at 95 °C until its core temperature reached 80 °C. The cooked meat was cooled in iced water until a core temperature of 15 °C was recorded. The cooked meat was then rested at 4 °C for at least 1 h before analysis. Cook loss was expressed as the difference, in percentage, between the weights before and after the meat was cooked. The cooked meat was cut into four rectangular (10 mm × 20 mm × 10 mm) and three cubic (10 mm × 10 mm × 10 mm) specimens, and subjected to texture analyses using a TA-XTi texture analyzer (Stable Micro Systems, Godalming, UK). The rectangular specimens were subjected to a shear test in which the texture analyzer was equipped with a V-shaped Warner Bratzler cutting blade. The cooked meat was sheared perpendicularly to the muscle fiber direction at a cutting speed of 1 mm/s test speed, 25–30 mm working distance and 0.2 N trigger force. As for the cubic specimens, the samples were double compressed to 40% of their initial height using a 25-kg loading cell connected to a 50-mm cylindrical aluminum probe. The test conditions were set as follows: 1 mm/s probe velocity, 1 s holding time and 0.1 N trigger force [34]. Shear force and hardness of the cooked meat were automatically calculated and reported by the Exponent software (Stable Micro Systems).

### 2.4. Absolute Gene Expression

Absolute mRNA abundances of superoxide dismutase 3 (*SOD3*), hypoxia-inducible factor 1 (*HIF-1A*), pyruvate dehydrogenase kinase (*PDK1*) and lactate dehydrogenase A (*LDHA*) genes were determined by droplet digital polymerase chain reaction (ddPCR) assay of cDNA produced from reverse-transcription reactions. The respective proteins encoded by those four genes play distinct roles in adaptive mechanisms when cells encounter oxidative stress [20]. The ddPCR primer sequences are shown in Table 1.

Total RNA was isolated from the 20-min postmortem muscle samples using TRizol™ Reagent (Life Technologies, Inc., Carlsbad, CA, USA). The isolated RNA was incubated with DNase I (Thermo Scientific, Inc., Rockford, IL, USA) according to the manufacturer’s instruction and subsequently purified using a GeneJet RNA Cleanup and Concentration Micro kit (Thermo Scientific, Inc. Rockford, IL, USA). Quantity and quality of total RNA were determined using a NanoDrop spectrophotometer (model 2000, Thermo Scientific, Inc., Wilmington, DE, USA). Total RNA (1.5 μg) was reversed transcribed into cDNA with oligo(dT) primer using an ImProm-II™ Reverse Transcription System kit (Promega Corporation, Madison, WI, USA). The amount of the synthesized cDNA was determined using a NanoDrop spectrophotometer.

The 20-μL ddPCR mixture comprised 1X EvaGreen^®^ supermix (Bio-Rad Laboratories, Inc., Hercules, CA, USA), 0.25 µM of each forward and reverse primer, and cDNA template as specified in Table 1. No template control was added in every run by replacing the cDNA template with an equal volume of nuclease-free water. The reaction mixture was loaded into a QX100™ droplet generator (Bio-Rad Laboratories, Inc.) according to the manufacturer’s instruction to generate water-in-oil droplet emulsion. The droplets were transferred to a 96-well plate, heat sealed and placed into a conventional thermocycler (model T100™, Bio-Rad Laboratories, Inc.). The reaction conditions were 95 °C for 5 min; followed by 40 cycles of 95 °C for 30 s, 58 °C for 1 min, and 4 °C for 5 min; and 90 °C for 5 min. After the amplification, the plate was transferred to a QX200™ droplet reader (Bio-Rad Laboratories, Inc.) where fluorescent signal intensity of the droplets was measured. The fluorescence amplitude threshold was set under the high amplitude droplet cluster to distinguish positive and negative droplets. The detected droplets were analyzed in copies per 20 µL reaction by QuantaSoft™ software (Bio-Rad Laboratories, Inc.) and divided by the amount of cDNA added to the reaction to obtain the absolute copy numbers per nanogram of template.

### 2.5. Statistical Analysis 

Statistical analysis was conducted using an SPSS Statistics for Windows, version 11.5 (SPSS, Inc., Chicago, IL, USA). The *t*-test for unequal sample sizes was used to evaluate the differences in means between non-WB and WB samples. The significant level was set at α = 0.05.

## 3. Results

Pectoralis muscle samples were obtained from broiler carcasses showing evidence of WB and a control group (non-WB). The initial inspection of sample compositions showed that the mean pH_20_ of the WB group was significantly lower than that of the non-WB group (*p* ≤ 0.05), whereas the lactate and glycogen contents were not significantly different (*p* > 0.05; Table 2).

Considering the chemical compositions of the meat samples (Table 3), the raw WB meat samples contained significantly greater moisture content but lower protein and ash contents compared with raw non-WB samples (*p* ≤ 0.05). However, on the basis of solid content (dry basis), the protein and fat contents of WB and non-WB were not significantly different (*p* > 0.05). Lactate was found to be lower in the WB meat samples compared with that of the non-WB samples (*p* ≤ 0.05). As for protein carbonyls and TBARS, the indicators for oxidation of protein and lipid, respectively, the WB samples exhibited a significantly higher level of protein carbonyls (*p* ≤ 0.05) indicating a greater degree of protein oxidation. However, the difference in the TBARS mean values between non-WB and WB meat samples was not significant (*p* > 0.05), suggesting that the extent of lipid oxidation was not different among the two sample groups.

Next, the amino acid compositions were compared between the WB and non-WB sample groups (Table 4). Among the essential amino acids, isoleucine (Ile), leucine (Leu) and valine (Val) were significantly lower in WB (*p* ≤ 0.05), whereas lysine (Lys) was significantly greater in WB (*p* ≤ 0.05). Among the non-essential amino acids, alanine (Ala), aspartic acid (Asp) and glutamic acid (Glu) were significantly lower in WB (*p* ≤ 0.05), whereas cysteine (Cys) and cystine (C-C) content were significantly higher in WB (*p* ≤ 0.05). 

Elements and trace elements detected in the breast meat samples are shown in Table 5. The WB breast samples contained significantly lower phosphorus and potassium (*p* ≤ 0.05), whereas the contents of aluminum, calcium, iron, sodium and sulfur were significantly higher in the WB breast samples (*p* ≤ 0.01).

The technological properties of the broiler breast samples are shown in Table 6. No difference in pHu was observed (*p* > 0.05). Focusing on the surface color, the mean L*-value of the raw WB meat was significantly greater (*p* ≤ 0.05), indicating that the raw WB meat appeared brighter compared with non-WB. The color difference (∆E) of 3.59 calculated from the L*a*b* values is greater than the difference value of 2.3 perceptible to the naked eye [35], indicating that a perceivable color distinction exists between the two groups. In addition, the raw WB meat exhibited a significantly higher degree of drip loss than the non-WB group (*p* ≤ 0.01). The comparisons of cooked meat samples showed that significantly less shear force was required to cut through WB samples (*p* ≤ 0.05), whereas no difference in hardness was observed between the two groups (*p* > 0.05).

**Figure 1:**
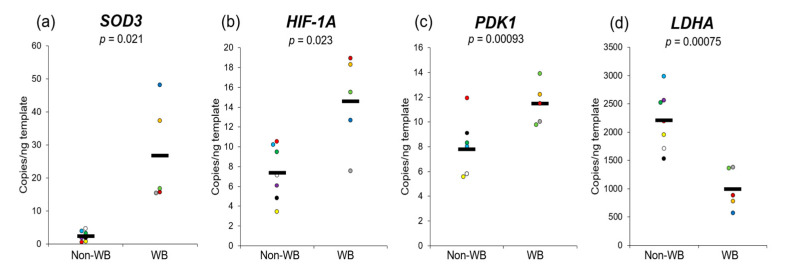
Absolute expression of (**a**) superoxide dismutase 3, extracellular (*SOD3*), (**b**) hypoxia-inducible factor 1; alpha subunit (*HIF-1A*), (**c**) pyruvate dehydrogenase kinase (*PDK1*) and (**d**) lactate dehydrogenase A (*LDHA*) in pectoralis major muscle of 7-week-old broilers unaffected (*n* = 7) and affected (*n* = 5) with WB abnormality. Each dot represents the absolute expression level of each sample in copies/ng template. The solid lines show mean values of each group.

The expression levels of genes in muscle tissue samples with functions related to oxidative stress were tested by ddPCR (Figure 1). The absolute abundances of *SOD3*, *HIF-1A* and *PDK1* were significantly greater in the WB group (3.9, 2.0, and 1.5-fold, respectively; *p* ≤ 0.05) compared with non-WB. In contrast, *LDHA* abundance in WB was 2.2-fold lower than that of non-WB (*p* ≤ 0.05).

## 4. Discussion

Development of WB myopathy markedly impacts the meat quality of broiler breasts. The effects of WB condition on the chemical composition found in this study agreed with previous findings [8,12,36]. The greater moisture content of WB-affected breast samples is potentially a consequence of fluid accumulation within the tissue due to inflammation of muscle tissue [6], or blood vessel [19]. WB-affected meat is also reported to exhibit decreased protein and increased fat contents [8,12,37], which are attributed to myodegeneration and the aberrant replacement of new muscle fibers with deposition of fat and connective tissues [6,8,38]. However, another study comparing the proximate composition of breast samples collected from Cobb broilers slaughtered at 44 d of age between non-WB and extremely severe WB samples found only a slight decrease in protein in WB and no differences in fat and collagen content [13]. In this study, the proportion of protein in the WB meat was decreased only on the wet basis, suggesting that the reduced protein content observed in the WB meat can be attributed to greater moisture content. As for fat, no differences in fat content either on the wet or dry basis were observed. The discrepancy might be due to the unevenly distributed WB lesions throughout the broiler breast [15]. Another explanation, that should be noted, was the small sample size and large variation among the biological samples, which reduced the statistical power and the probability of detecting an effect.

pH is one of the crucial parameters determining meat quality. Previous studies reported elevated ultimate pH (pHu) in WB meat compared with that of normal breast meat [20,39,40,41,42]. However, in this study no difference in pHu was observed, but a significantly lower pH_20_ was observed for WB samples. Although there is no clear explanation for this discrepancy, several factors are known to affect early postmortem pH in the animals, including physical activities [43], ATPase-related cellular activities [44] and stress [45] before death. For pHu, this value mainly depends on muscle glycogen content, which refers to the rate of post-mortem glycolysis [43] and the status of energy-generating and utilization pathways [44]. We observed no significant difference in glycogen and lactate contents between WB and non-WB, which could explain why no difference in pHu between the two groups was observed. Moreover, it has been speculated that the carbohydrate flow from glycolysis is rerouted to other metabolic pathways in order to mitigate muscle inflammation in WB [44].

A number of studies highlighted the negative effects of WB myopathy on technological indices of the meat [8,11,39,46,47]. In this study, the aberrantly lighter surface color and greater drip loss in the WB samples were in agreement with previous reports [40,47]. The pale appearance of the WB samples could be due to the altered light scattering caused by edema in WB meat [48]. The yellowness (b* value) was previously reported to be higher in WB than in non-WB [49] and speculated to be the result of a strong fibrotic response [46]. Our current data indicated that the b* value of the WB (2.99 ± 0.99) also tended to be higher than that of the non-WB (0.53 ± 0.34) despite the probability (*p* = 0.065).

An increase in the hardness of cooked WB meat has been reported in other studies [9,46,50,51], with a corresponding increase in collagen content in one study [8]. In this study, no difference in hardness was observed between the cooked WB and non-WB samples; however, shear force was found to be lower in the former (Table 6). These findings are in agreement with Sanchez-Brambila et al. [52], who reported that among the broiler breast samples stored at 4 °C for 5 days, cooked WB showed a lower degree of shear force and no difference in hardness compared with non-WB. The lower shear force of WB in this study might reflect the disintegration of muscle fibers usually found in birds with gigantic muscle fibers [52,53,54]. No difference in hardness between cooked WB and non-WB has also been reported in other studies [40,55]. In the study of Soglia et al. [55], either hardness or the values of shear force of cooked WB samples was different from those of non-WB ones. The results were proposed to be related with heat denaturation and solubilization of collagen during cooking [55]. In addition, the comparable content of hydroxyproline, the major component of collagen, as determined using GC–MS (Table 4) might partly be responsible for the indifferent hardness between the two groups. Moreover, heterogeneity of WB lesions throughout the breast [15] and interstitial connective tissue in muscle [56,57] might also be responsible for the variation in hardness and shear force among samples.

Concerning the nutritional quality, development of WB potentially lowered nutritional values of the chicken breast meat in terms of protein quality and mineral profile. WB meat exhibited a lower protein-to-fat ratio, altered amino acid profile and potentially reduced protein quality through oxidation. Limited oxygen availability in the pectoralis major muscle of WB-affected animals has been reported [15,19,37,58,59], which is potentially due to insufficient blood supply [15]. Under this condition, ROS can be generated and accumulate to react with lipids and proteins. In this study, an increased *SOD3* abundance was observed in the WB-affected muscle (Figure 1), suggesting a protective antioxidant response to high ROS. Although no significant difference in lipid oxidation as indicated by TBARS was observed in this study, a greater proportion of cystine (C-C) was detected in WB meat based on data of GC–MS and protein carbonyls. This result suggested that the proteins in the abnormal breast samples underwent oxidation, resulting in the formation of disulfide bridges between sulfhydryl groups and protein carbonylation to a greater extent, which is consistent with the findings of Soglia et al. [60]. In addition, global or local disorder in the protein structure can expose and make the peptide backbone vulnerable to oxidation through the formation of α-carbon backbone radicals [61], which ultimately leads to peptide bond cleavage and formation of carbonyl groups. Taken together, the greater protein carbonyl and cystine contents along with a reduced proportion of amino acids in WB suggest increased ROS-induced protein oxidation in WB.

Protein carbonyls are generally used as oxidative stress biomarkers [62,63]. ROS-induced carbonylation of protein is an irreversible modification that frequently occurs at the side chains of cysteine, histidine and lysine residues [64]. The carbonyl derivatives possess the ability to react with side chains of other amino acids, particularly tyrosine and threonine, further reducing availability of essential amino acids. Consequently, protein oxidation can result in intramolecular and intermolecular polymerization and aggregation and, in severe cases, breakage of the polypeptide backbone. Polymerization and aggregation of the oxidized proteins can negatively affect WHC [65], which corresponds with the increased drip loss observed herein and in previous reports [8,18,36,47]. Due to lower protein content and protein oxidation in WB meat, a greater proportion of water can exist as an extra-myofibrillar (free) water fraction [49,60], which primarily contributes to drip loss upon application of external forces, e.g., gravity. The lower WHC in WB has also been linked with the replacement of fat and connective tissues and muscle fiber damage [8,60]. Our findings provide evidence that protein oxidation could be another factor affecting the ability of protein to hold water in the WB samples.

The interactions between lipid and protein oxidation in meat are still not fully understood, although lipid oxidation products could play a role in promoting protein oxidation. Soyer et al. [66] reported an increase in TBARS concomitant with increased protein carbonyls and reduced sulfhydryls in chicken breast meat stored frozen for 6 months. Rodan et al. [67] monitored primary and secondary products from lipid oxidation and total protein carbonyls in lamb loin cooked at different temperatures and for different durations. Their results indicated an initial increase in TBARS up until 6 h of sous-vide cooking, followed by a decrease to the basal level. The total amount of protein carbonyls, however, steadily increased throughout cooking time at all cooking temperatures tested. Rodan et al. [67] suggested that malondialdehyde, a dicarbonyl end product of lipid peroxidation, was prone to react with other compounds, particularly protein side chains, leading to decreased TBARS values but increased protein carbonyls after prolonged cooking. Unlike malondialdehyde, the reactivity of the carbonyl groups on the oxidized protein may be limited by steric hindrance and low mobility of high-molecular weight molecules, resulting in accumulation of protein carbonyls. This scheme can explain the greater protein carbonyl content of WB meat observed in this study.

In addition to the reduced protein quality, WB meat showed a different mineral profile. The ash content of the WB samples was significantly lower than that of the non-WB, in agreement with previous reports [8,12,18]. Furthermore, we found significantly elevated contents of calcium and sodium in WB in agreement with others [8,18,46], which has been linked with an ion imbalance in the WB muscle [18]. In general, sodium is presented within poultry meat at a small amount; hence, the meat is recommended for the patients with hypertension [3]. Dysregulated calcium metabolism could interfere with muscle membrane integrity and lead to myodegeneration in WB-affected animals [58]. Increased iron might partly be a consequence of hemorrhagic lesions, the common characteristics of WB myopathy [29], or the high myoglobin content of WB muscle. Zambonelli et al. [18] reported approximately five-fold increased transcript abundance of myoglobin in the pectoralis major of WB broilers in comparison with that of unaffected birds, which was hypothesized to be stimulated by hypoxia [68]. The concentration of iron-containing pigments in meat plays a crucial role in the catalysis of lipid peroxidation [23]. Therefore, WB meat is more susceptible to oxidation because of its greater iron content. In addition, the WB samples tended to have lower contents of copper, zinc and selenium (Table 5), which are the three essential minerals expected to receive from the consumption of poultry meat [3]. Those three minerals are also the crucial components for antioxidant enzymes, e.g., superoxide dismutase and glutathione peroxidases, involved in physiological protective systems against oxidative stress in poultry [69]. Additionally, Estevéz et al. [70] addressed the protective effects of magnesium in reducing WB incident and severity and the oxidation of protein and lipid within broiler breast [70]. Consumption of WB chicken meat with the observed levels of alteration in the mineral profiles might not directly pose risks of detrimental health effects to individuals in the general population. However, among the susceptible groups such as the patients with hypertension, the changes in mineral content, particularly sodium, could exert a significant health impact to their health condition [71]. It is worth noting that the XRF technique employed for quantitative elemental determination in meat samples in this study and others [72,73,74] determine the element contents based on surface scanning; therefore, an inhomogeneous distribution of minerals in the prepared samples can lead to the over- or underestimated contents of certain elements. In addition, we suspect that the elevated aluminum content in WB arose from contamination from a pan made from aluminum foil used during determination of moisture content. A previous report noted contamination from aluminum foil during cooking [75].

Cellular hypoxia is proposed to be the major factor responsible for the development of WB myopathy [15,20,55,76]. In response to hypoxic stress, the HIF-1 complex induces transcription of hypoxia-responsive elements leading to suppression of mitochondrial pyruvate catabolism and oxygen consumption, and the switch of glucose metabolism from oxidative phosphorylation to anaerobic glycolysis. An increased level of *HIF-1A* transcript was detected in WB, which is consistent with our previous study [20]. The increased expression of *PDK1* is consistent with it being an activated target of HIF-1A. PDK1 indirectly inhibits the entry of pyruvate to the TCA cycle, leading to attenuation of oxidative phosphorylation and ROS [77]. WB exhibited a lower abundance of *LDHA* transcript (Figure 1), which corresponded with the low lactate content observed in WB meat (Table 3). The decreased *LDHA* agreed with previous studies [20,78], providing further support to the suggestion that lactate homeostasis is aberrant in WB [79] or the response to hypoxia in WB muscle occurs through alternative pathways [80].

Recent studies of Salles et al. [81] and Carvalho et al. [82] revealed association of oxidative damage with white striping (WS) abnormality, another emerging growth-induced myopathy of wide concern in the broiler industry [5]. Salles et al. [78] reported that severe WS showed higher levels of ROS and oxidative protein products accompanied with increased activities of glutathione S-transferase. Carvalho et al. [82] reported evidence of oxidative damage in severe WS, including increased TBARS, allysine and Schiff bases and impaired activities of the antioxidant enzymes catalase, glutathione peroxidase and SOD. In addition, L-lactate dehydrogenase, creatine kinase and pyruvate kinase proteins were downregulated in severe WS. Those studies together with the current findings indicate that aberrant oxidative status is a common feature of growth-induced myopathies in broilers.

## 5. Conclusions

The WB myopathy affects not only the physical appearance, but also the nutritional quality of chicken breasts. This study identified other features affected by WB, including moisture and protein contents, essential amino acid and mineral profiles, water holding capacity and oxidation at physiological and transcriptional levels. The current findings suggest that oxidative stress in WB-affected broilers exerts negative impacts on both the technological properties and nutritive values of the chicken breast. Further investigation regarding the effects of WB myopathy on protein digestibility is of interest to obtain a better understanding on the effects of chicken myopathy on human health.

## Figures and Tables

**Table 1:** Forward and reverse primers of the genes analyzed using ddPCR.

NCBI Accession	Gene ID	Sequence (5’→3’)	Amplicon Size (bp)	Template ^1^ (ng)
XR_001466725.2	*HIF-1A*	F: ATCAGAGTGGTTGTCCAGCAG R: CAGTCCAAGCCCACCTTACT	111	25
NM_205284.1	*LDHA*	F: TTCTCTGCCAGCTGAATAGCTT R: CGGGTCATTGTCTTGTTGCAT	200	1
NM_001031352.3	*PDK1*	F: TGCCAAGCAGTGAGCCAAAG R: AACTCCCTTCACATGACACACAT	97	10
XM_015285700.1	*SOD3*	F: TACAAACCCAACCTCTTCGC R: GTTATTGCCCTTGCCCATGT	102	10

^1^ cDNA template (ng) in 20 μL ddPCR reaction, obtained from preliminary optimization. *HIF-1A* = hypoxia-inducible factor 1 alpha subunit, *LDHA* = lactate dehydrogenase A, *PDK1* = pyruvate dehydrogenase kinase isozyme 1, *SOD3* = superoxide dismutase 3.

**Table 2:** The 20-min postmortem pH, glycogen and lactate content of pectoralis major muscles.

Composition	Non-WB	WB	*p*-Value
pH_20_	6.86 ± 0.04	6.71 ± 0.03	0.012
Glycogen (mg/g muscle)	2.41 ± 0.40	1.90 ± 0.39	0.332
Lactate (mg/g muscle)	2.55 ± 0.14	2.81 ± 0.21	0.217

The data are presented as mean ± standard error. non-WB = non-wooden breast (*n* = 7) and WB = wooden breast (*n* = 5).

**Table 3:** Chemical composition and oxidative indices of broiler breast meats.

Composition	Non-WB	WB	*p*-Value
Moisture (%)	74.76 ± 0.55	77.86 ± 0.60	0.004
Protein (% wet basis)	22.12 ± 0.22	18.67 ± 0.57	0.002
Fat (% wet basis)	1.62 ± 0.05	1.52 ± 0.19	0.667
Protein (% dry basis)	87.92 ± 2.27	84.38 ± 1.49	0.222
Fat (% dry basis)	6.41 ± 0.21	6.81 ± 0.66	0.583
Ash (% wet basis)	1.16 ± 0.03	1.05 ± 0.02	0.012
Lactate (mg/g meat)	7.87 ± 0.51	6.18 ± 0.35	0.022
TBARS (μmol malondialdehyde/kg meat)	1.15 ± 0.11	1.23 ± 0.06	0.593
Protein carbonyls (nmol/mg protein)	0.79 ± 0.05	1.09 ± 0.10	0.029

The data are presented as mean ± standard error. non-WB = non-wooden breast (*n* = 7). WB = wooden breast (*n* = 5). TBARS = Thiobarbituric acid reactive substances.

**Table 4:** Amino acid profile (% of total amino acids) of broiler breast meats.

Amino Acid		Non-WB	WB	*p*-Value
Essential amino acids				
Arginine	Arg	4.77 ± 0.53	4.08 ± 0.23	0.098
Histidine	His	3.14 ± 0.18	3.08 ± 0.17	0.721
Isoleucine	Ile	4.93 ± 0.16	4.28 ± 0.31	0.020
Leucine	Leu	7.92 ± 0.20	7.33 ± 0.25	0.015
Lysine	Lys	7.70 ± 0.37	9.30 ± 0.81	0.025
Methionine	Met	3.73 ± 0.12	3.58 ± 0.14	0.229
Phenylalanine	Phe	4.32 ± 0.10	4.11 ± 0.12	0.055
Threonine	Thr	5.78 ± 0.12	5.71 ± 0.07	0.495
Tryptophan	Trp	0.80 ± 0.04	0.71 ± 0.09	0.186
Valine	Val	5.08 ± 0.15	4.72 ± 0.09	0.008
Non-essential amino acids				
Alanine	Ala	6.28 ± 0.08	6.09 ± 0.07	0.019
Aspartic acid	Asp	8.31 ± 0.33	7.53 ± 0.16	0.008
Cysteine	Cys	2.41 ± 0.08	2.75 ± 0.14	0.010
Glutamic acid	Glu	14.12 ± 0.39	13.09 ± 0.15	0.003
Glycine	Gly	4.14 ± 0.22	4.26 ± 0.06	0.418
Hydroxyproline	Hyp	1.33 ± 0.13	1.17 ± 0.12	0.181
Proline	Pro	5.13 ± 0.40	4.82 ± 0.19	0.298
Serine	Ser	4.01 ± 0.10	4.05 ± 0.03	0.548
Tyrosine	Tyr	3.07 ± 0.12	3.09 ± 0.07	0.761
Cystine	C-C	3.05 ± 1.25	6.23 ± 0.65	0.005

The data are presented as mean ± standard error. non-WB = non-wooden breast (*n* = 7). WB = wooden breast (*n* = 5).

**Table 5:** Elements and trace elements (mg/kg meat) of broiler breast meats.

Mineral	Non-WB	WB	*p*-Value
Aluminum (Al)	0.0 ± 0.0	50.8 ± 20.8	<0.001
Calcium (Ca)	55.9 ± 13.6	86.1 ± 39.9	0.01
Chromium (Cr)	5.7 ± 0.6	5.0 ± 0.6	0.69
Copper (Cu)	3.6 ± 3.7	2.2 ± 0.4	0.46
Iron (Fe)	8.6 ± 0.9	9.0 ± 1.2	0.01
Magnesium (Mg)	351.2 ± 68.9	284.9 ± 42.5	0.24
Phosphorus (P)	2219.2 ± 78.0	1958.2 ± 100.2	0.02
Potassium (K)	3153.9 ± 114.2	2758.4 ± 278.6	0.04
Selenium (Se)	0.3 ± 1.0	0.0 ± 0.0	0.35
Sodium (Na)	392.1 ± 70.1	545.4 ± 160.3	0.001
Sulfur (S)	11.1 ± 11.2	57.3 ± 30.1	<0.001
Zinc (Zn)	14.3 ± 2.0	15.0 ± 2.7	0.06

The data are presented as mean ± standard error. non-WB = non-wooden breast (*n* = 7). WB = wooden breast (*n* = 5).

**Table 6:** Technological properties of broiler breast meats.

Property	Non-WB	WB	*p*-Value
pHu	6.01 ± 0.06	6.04 ± 0.06	0.717
L*-value	52.21 ± 0.75	54.82 ± 0.54	0.018
a*-value	2.60 ± 0.23	2.66 ± 0.17	0.821
b*-value	0.53 ± 0.24	2.99 ± 0.99	0.065
Drip loss (%)	0.89 ± 0.14	2.04 ± 0.23	0.004
Cook loss (%)	19.54 ± 1.95	22.81 ± 3.04	0.396
Shear force (N)	39.79 ± 2.68	26.87 ± 4.09	0.032
Hardness (N)	27.48 ± 0.86	27.02 ± 3.17	0.894

The data are presented as mean ± standard error. non-WB = non-wooden breast (*n* = 7). WB = wooden breast (*n* = 5). L*, a*, and b*-value indicate lightness, greenness/redness and blueness/yellowness, respectively, in CIE L*a*b* color system.
